# Divanillin-Based Aromatic Amines: Synthesis and Use as Curing Agents for Fully Vanillin-Based Epoxy Thermosets

**DOI:** 10.3389/fchem.2019.00606

**Published:** 2019-09-06

**Authors:** Etienne Savonnet, Cedric Le Coz, Etienne Grau, Stéphane Grelier, Brigitte Defoort, Henri Cramail

**Affiliations:** ^1^Univ. Bordeaux, CNRS, Bordeaux INP, LCPO, UMR 5629, Pessac, France; ^2^ArianeGroup, St-Médard-en-Jalles, Bordeaux, France

**Keywords:** di-vanillin, amines, curing agent, thermoset, epoxy

## Abstract

Bio-based aromatic diamines from vanillin substrate were successfully synthesized and characterized. These amines, i.e., methylated divanillylamine (MDVA) and 3,4-dimethoxydianiline (DMAN), were then tested as curing agents for the design of bio-based epoxy thermosets. The epoxy thermosets obtained from these novel vanillin-based amines exhibited promising thermomechanical properties in terms of glass transition temperature and char residue.

## Introduction

Epoxy thermosets are the products of the reaction between epoxy-based monomers or prepolymers with curing agents. The cross-linking agents are significant in terms of mass fraction as the latter can represent up to 50% of the formulations. Many efforts are currently undertaken to develop bio-based alternatives to traditional epoxy monomers derived from fossil resources and, in particular, bisphenol-A (Kumar et al., 2018). With similar objectives, some studies already reported the synthesis of bio-based anhydride- or acid-type curing agents from renewable resources, such as vegetable oils (Roudsari et al., 2014), rosin (Wang et al., 2008, 2009; Liu et al., 2009; Qin et al., 2014a), terpens Takahashi et al., 2008, tannins Pizzi, 2008; Shibata and Nakai, 2010, or lignins (Qin et al., 2014b).

Moreover, aliphatic amine curing agents have also been reported from substrates originating from biomasses such as terpenes (Keim and Roeper, 1981; Keim et al., 1983; Garrison and Harvey, 2016), lignin (Fache et al., 2014), cardanol (Thiyagarajan et al., 2011; Shingte et al., 2017) derived from cashew oil or vegetable oils (Stemmelen et al., 2011; Hibert et al., 2016; Samanta et al., 2016). Very recently, amines have been synthesized from vanillin (Mora et al., 2018) by NH_3_ addition onto diglycidylated vanillin alcohol. When cured with classical DGEBA, a Tg of 72°C of the so-formed epoxy thermoset was obtained.

Despite all these works, the development of bio-based fully aromatic amine-type curing-agents leading to high Tg epoxy thermosets is still an unmet challenge. In this way, there is a growing interest to find bio-based reactive amines leading to epoxy thermosets with high thermomechanical properties.

Vanillin is a very interesting candidate because it is one of the non-hazardous aromatic compounds industrially available from biomass (Pinto et al., 2012). From vanillin, we developed an efficient C–C coupling reaction through enzymatic catalysis leading to highly pure divanillin substrate (Kobayashi and Makino, 2009; Llevot et al., 2015, 2016). More recently, we have developed a palette of epoxy monomers derived from divanillin, which demonstrated to be valuable, and realistic alternative to DGEBA-based epoxy thermosets (Savonnet et al., 2018). In the following of our previous investigations, this article presents two synthetic pathways to prepare primary aromatic amines from divanillin as starting material. These polyfunctional amines were then used as curing agents for the synthesis of epoxy thermosets.

## Synthesis of Bio-based Amines From Vanillin Derivatives

Herein, two synthetic pathways have been identified for the synthesis of bio-based aromatic amines through the reduction of oxime and the acyl azide rearrangement moieties, leading to bis-benzylamine and bis-aniline moieties, respectively.

**Scheme 1:**
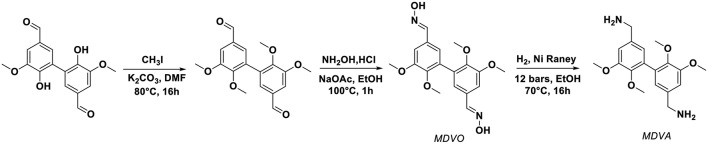
Synthesis of methylated divanillylamine from divanillin.

**Figure 1:**
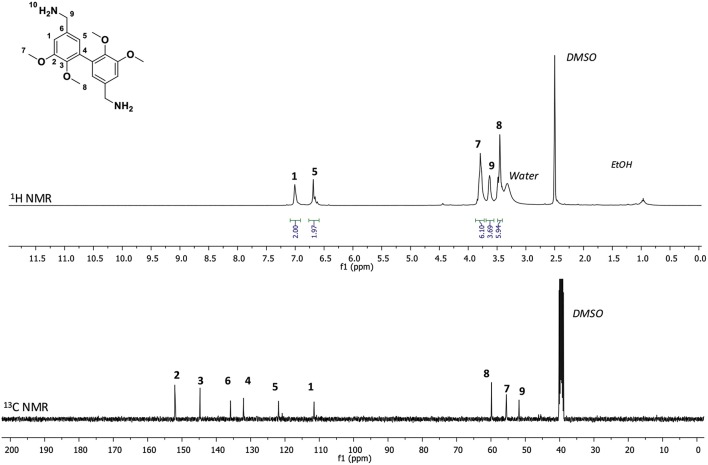
^1^H and ^13^C NMR spectroscopy of methylated divanillylamine (MDVA) in DMSO-d6.

The divanillin displays two aldehyde functions which can undergo chemical reaction to get divanillyloxime. A synthetic pathway adapted from literature has been developed and is depicted on Scheme 1 (Liu et al., 2011; Fache et al., 2015). First, the alkylation of phenol moiety was performed in the presence of iodomethane and a weak base leading to methylated divanillin in a quantitative yield (>95%). Then, the oximation step consisted in the reaction of aldehyde functions with hydroxylamine hydrochloride in the presence of sodium acetate to yield methylated divanillyloxime (MDVO) (>95%). The structure of this intermediate was confirmed by ^1^H and ^13^C NMR spectroscopies (Figure S1). The appearance of a signal at 3.67 ppm was attributed to the methylated phenols and new signals at 8.10 and 11.13 ppm were attributed to the oxime moieties of methylated divanillyloxime (MDVO). Finally, the so-formed oxime was then reduced into methylated divanillylamine (MDVA) by hydrogenation. The reaction was performed during 16 h at 70°C under 12 bars of H_2_ pressure in the presence of Nickel Raney in ethanol. Reduction of the oxime yielded a pale orange solid with a melting temperature of 69°C (DSC). ^1^H NMR spectrum of this orange solid demonstrated the disappearance of oxime signals at 8.10 and 11.13 ppm and the appearance of a new signal at 3.63 ppm, corresponding to the benzylic-protons of the amine (Figure 1). ^13^C NMR spectrum exhibited also a shift of the alpha-carbon of the oxime from 147.81 to 51.62 ppm, which confirmed the reduction of oxime moieties. The attribution of the signals was also confirmed by HSQC NMR spectroscopy. In addition, the MDVA was also characterized by FTIR. Figure S2a shows the infra-red spectra of MDVO and MDVA from which the decrease of O-H stretching signal at 3,227 cm^−1^ and the disappearance of N-O stretching at 945 cm^−1^ were observed and corresponding to the oxime moieties. Besides, ninhydrin test (Friedman, 2004), which reveals amino groups, confirmed the presence of amine moieties on MDVA (Figure S2b).

**Scheme 2:**
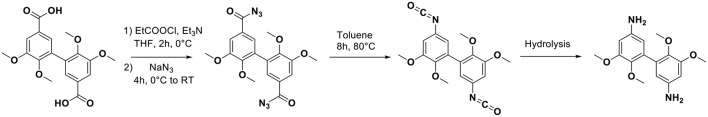
Synthesis of 3,4-dimethoxydianiline (DMAN) from methylated divanillic acid.

The second pathway is based on the Curtius rearrangement. It involves the synthesis of an acyl azide intermediate. Usually carboxylic acids are precursors of acyl azides. As previously described (Kobayashi and Makino, 2009; Llevot et al., 2015, 2016), methyl vanillate can easily be dimerised into the corresponding dimer and hydrolyzed into the diacid. From divanillic acid, the sequential synthetic pathway is summarized in Scheme 2. All the intermediates were obtained without any further purification steps, unless mentioned. ^1^H and ^13^C NMR spectroscopies were performed to confirm the structure of the synthesized products. Methyl divanillate was first alkylated using the same procedure described previously and obtained in a high yield (>80%). Thereafter, the hydrolysis of the methylated diester with sodium hydroxide yielded the diacid (>90%). The disappearance of the methyl ester protons at 3.38 ppm and the appearance of acid proton signals of carboxylic acid at 9.20 ppm were attributed to the formation of the methylated divanillic acid. Methylated divanillic acid was then converted into acyl azide in a two-step reaction. Ethyl chloroformate was first reacted with the acid to form *in situ* an acyl chloride and sodium azide was then added to the mixture, yielding the corresponding acyl azide (>60%). Finally, the di-isocyanate was obtained in good yield (>80%) by simply heating the azide compound in dry toluene. The synthesis of these latter compounds was confirmed by ^1^H NMR spectroscopy (Figure S3).

In addition, acyl azide structure was confirmed by the disappearance of carboxylic signal at 9.20 ppm and finally the shift of the aromatic protons from 7.61 and 7.55 to 6.65 and 6.58 ppm was attributed to the so-formed di-isocyanate. These compounds were also characterized by FTIR spectroscopy (Figure S4). Infrared spectra exhibited characteristic signal wavelengths of carboxylic acid at 1,720 cm^−1^ (-C = O) and 2,500–3,300 cm^−1^ (O-H), acyl azide at 2,140 cm^−1^ (-N_3_), and isocyanate at 2,278 cm^−1^ (-N = C = O).

**Figure 2:**
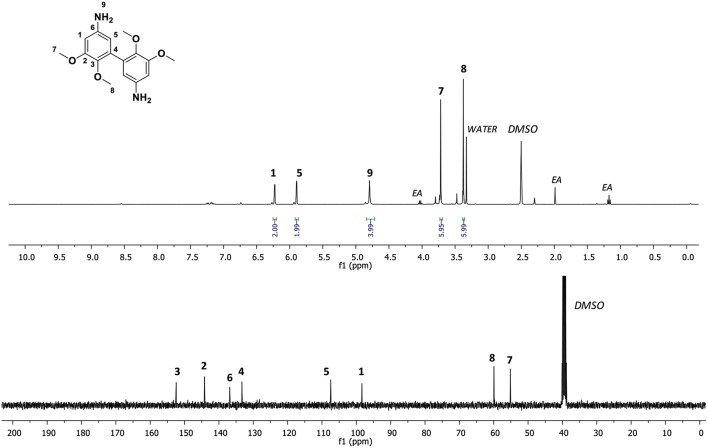
^1^H and ^13^C NMR spectroscopy of 3,4-dimethoxydianiline (DMAN) in DMSO-d6.

Finally, 3,4-dimethoxydianiline (DMAN) was recovered by hydrolysis in basic conditions of the corresponding di-isocyanate. After extraction with ethyl acetate and washing with water a mixture of brown and white solids was obtained. However, ^1^H NMR spectroscopy of the reaction mixture revealed the presence of a by-product (Figure S5). These additional signals could be attributed to the formation of ureas, resulting of the side reaction of amine with the isocyanate or to the oxidation of the amines. Nevertheless, a small fraction was isolated as a white solid corresponding to the DMAN and characterized by ^1^H and ^13^C NMR spectroscopy (Figure 2). ^1^H NMR spectroscopy displayed proton signals of aromatic rings, amine moieties and alkylated hydroxyl groups at 6.23, 5.90, 4.79, 3.72, and 3.38 ppm, respectively. In addition, the disappearance of the C9 carbon signals of isocyanate at 124.71 ppm, confirmed the obtention of the targeted diamine. DMAN was also characterized by FTIR spectroscopy (Figure S6). The following spectrum confirmed the absence of the isocyanate bands (-N = C = O) at 2,278 cm^−1^ and the appearance of amino groups signals at 1,610, 3,240, 3,370, and 3,471 cm^−1^. In conclusion, the Curtius rearrangement permitted to synthesize the 3,4-dimethoxydianiline from the acyl azide intermediate derived from the methyl divanillate. However, the last step of the synthesis was tedious and only a small amount of the desired product was identified as the diamine. Further investigations and optimizations in the hydrolysis step of the isocyanate substrate are required to recover the bio-based diamine in a better yield.

## Synthesis of Fully Vanillin-based Epoxy Thermosets

The previously synthesized di-amines, i.e., methylated divanillylamine (MDVA) and 3,4-dimethoxydianiline (DMAN) were used as curing agents for the synthesis of epoxy thermosets.

The DGEBA epoxy monomer was stirred vigorously with a stoichiometric amount of MDVA. The thermomechanical properties of the so-formed network were characterized by DSC and TGA. Bio-based thermoset was then compared with DGEBA/IPDA epoxy system. Results are summarized in the Table S1 and Figure S7. Unfortunately, the characterization of bio-based epoxy networks, using DSC, showed a weak exothermic peak (ΔH = 105 J/g for DGEBA/MDVA vs. 430 J/G for DGEBA/IPDA), corresponding to the reaction of amine with epoxy. In addition, no clear glass transition temperature was observed with this network (Figure S8). One hypothesis to explain this result could be the poor homogeneity of the mixture between DGEBA and the solid MDVA—MDVA melts at 40°C (DSC)-, which impaired the stoichiometric ratio.

DGEBA epoxy monomer was also cured with bio-based DMAN. However, in view of the small amount recovered, crude DMAN was used for the curing reaction. Thermomechanical properties of the network formed were then determined and compared with DGEBA networks cured with 4,4′-Diaminodiphenyl sulfone (DDS). Results are summarized in Table 1 and Figure S8b. In comparison with conventional amine hardener DDS, epoxy network cured with DMAN exhibited similar properties. Indeed, glass transition temperature of thermoset cured with DMAN displayed a value 30°C below the networks obtained with DDS, i.e., 176°C. This difference can be explained by the presence of by-product compounds, which could impair the stoichiometric ratio. Interestingly, despite the presence of the by-product, the bio-based amine enabled to increase the char yield residue up to 28% (Figure S8). This feature could be explained by the C-C bonding between the two aromatic rings of DMAN, which could favor the formation of char and thus increase the residual content. Another important difference between DDS- and DMAN-based epoxy thermosets is the higher reactivity of DDS.

**Table 1:** Thermomechanical properties of DGEBA and TetraGEDVA cured with DDS and DMAN.

**Epoxy/hardener**	**T_onset_(^°^C)**	**T_Exo_(^°^C)**	**ΔH(J.g^−1^)**	**ΔH(kJ.mol^−1^)**	**Tg(^°^C)**	**Char_900_(%)**
DGEBA/DDS	184	226	355	83	204	16
DGEBA/DMAN	99	153	393	95	176	28
TetraGEDVA/DDS	165	208	459	89	-	48
TetraGEDVA/DMAN	110	193	183	39	212	48

At last, a bio-based polyglycidylether, tetraglycidylether of divanillyl alcohol (TetraGEDVA), (Savonnet et al., 2018) was cured with crude DMAN. In this way, a fully bio-based epoxy thermoset was successfully obtained. Such bio-based epoxy network exhibited promising thermomechanical properties as attested by the glass transition temperature of 212°C and the char residue of 48% (Figure S8). However, the enthalpy of polymerization is much lower than the enthalpy of the reaction between TetraGEDVA and DDS. Again, the presence of undefined compounds in crude DMAN and the inhomogeneity of the mixture could explain this difference.

## Conclusion

In summary, the synthesis of bio-based curing agent from divanillin derivatives was investigated. Two routes were chosen to achieve the synthesis of diamines. The first one consisted in the reduction of divanillyloxime obtained from divanillin. The synthesis yielded methylated divanillylamine (MDVA) and the thermomechanical properties of thermosets obtained from epoxy precursors cured with MDVA were investigated. DGEBA epoxy prepolymers were thus cured with MDVA and the networks obtained were compared with the conventional DGEBA/IPDA system. Unfortunately, the characterization by DSC showed a weak exothermic peak, corresponding to the reaction of amine with epoxy, but no clear glass transition temperature could be observed with this system.

Then, the 3,4-dimethoxyaniline (DMAN) was synthesized using the oxidative rearrangement of Curtius. From methyl divanillate and through the synthesis of acyl azide and isocyanate intermediates, the hydrolysis of this latter yielded 3,4-dimethoxyaniline (DMAN). However, the hydrolysis step was tedious and the corresponding amine failed to be isolated efficiently. Nevertheless, crude DMAN was used as curing agent in the polyaddition reaction with DGEBA. Interestingly, the so-formed semi-biobased thermoset exhibited a glass transition temperature of 176°C against 204°C for the conventional DGEBA/DDS system. Moreover, the DMAN permitted to double the char content of the network comparing to DGEBA/DDS network cured in the same conditions. In addition, a fully bio-based epoxy thermoset was obtained by curing tetraglycidylether of divanillyl alcohol (TetraGEDVA) with DMAN. The thermomechanical properties obtained were promising as the epoxy thermoset exhibited a glass transition temperature of 212°C and a char residue of 48%.

Finally, new amine-type curing agents from vanillin-based starting material was successfully attempted and characterized. However, some further optimizations in the synthetic pathways described are still necessary to have a better appreciation of the potential of these new bio-based aromatic diamines in the course of thermoset designs.

## Data Availability

All datasets generated for this study are included in the manuscript/Supplementary Files.

## Author Contributions

ES performed the synthesis of the chemicals and performed the polymerization experiments. CL performed the thermo-mechanical analysis. EG, SG, BD, and HC contributed equally to the supervision of the project.

### Conflict of Interest Statement

BD was employed by ArianeGroup company. The remaining authors declare that the research was conducted in the absence of any commercial or financial relationships that could be construed as a potential conflict of interest.
