# Supplementary material for: Divanillin-Based Aromatic Amines: Synthesis and Use as Curing Agents for Fully Vanillin-Based Epoxy Thermosets
Source: Front Chem. 2019 Sep 6;7:606. doi: 10.3389/fchem.2019.00606 (PMC6743216; doi:10.3389/fchem.2019.00606)
Supplement: Supplementary file 1 [file Table_1.DOCX]

Divanillin-based aromatic amines: synthesis and use as curing agents for fully vanillin-based epoxy thermosets

Etienne Savonnet^1,2^ , Cedric Le Coz^1^, Etienne Grau^1^, Stéphane Grelier^1^*, Brigitte Defoort^2^, Henri Cramail^1^*

^1^Univ. Bordeaux, CNRS, Bordeaux INP, LCPO, UMR 5629, F-33600, Pessac, France

^2^ArianeGroup, Rue du général Niox, St-Médard-en-Jalles, 33160, France

*E-Mail: cramail@enscbp.fr; web: [www.lcpo.fr](http://www.lcpo.fr/)

This supporting information contains 9 figures over 13 pages.

**Experimental Section*:***

**Materials**

Laccase from Trametes versicolor, benzyltriethylammonium chloride (99 %), bisphenol-A diglycidylether (D.E.R ™ 332), hydroxylamine hydrochloride (99%), 4,4’-Diaminophenyl sulfone (97 %), hydrochloric acid (37,5 %), iodomethane (99 %), ethylchloroformate were purchased from Sigma-Aldrich. Vanillin (99 %), methyl vanillate (99 %) were purchased from Acros.

Potassium carbonate (99 %), triethylamine (99 %), potassium hydroxide (pellet) and sodium hydroxide (pellet) were purchased from Fisher.

TetraGEDVA was synthesized according to reported procedure.^[[1]](#footnote-1)^

All products and solvents (reagent grade) were used as received, unless mentioned explicitly.

***Procedure for dimerization of phenols***

A solution of vanillin (5mmol) in acetone (20 mL) was added to NaOAc buffer (180 mL, 0.1 M, pH 5.0). The solution was saturated in O_2_ for 5 min. Laccase from *Trametes versicolor* (20 U, 12.4 mg) was added and the reaction was stirred at room temperature for 24 h. The precipitate was filtered off the solution and the product dried overnight at 80 °C under vacuum. Yield: 90%


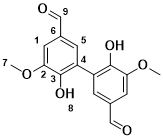


^1^H NMR (400 MHz, DMSO, δ (ppm)): δ 9.69 (s, H_9_), 7.57 (d, H_1_), 7.16 (d, H_5_), 3.76 (s, H_7_).

^13^C NMR (400 MHz, DMSO, δ (ppm)): δ 191.62 (s, C_9_), 150.88 (s, C_3_), 148.61 (s, C_2_), 128.64 (s, C_6_), 128.21 (s, C_4_), 125.02 (s, C_5_), 109.6 (s, C_1_), 56.25 (C_7_).

The same procedure was applied for methyl vanillate. Yield: 90%


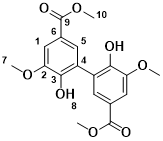


^1^H NMR (400 MHz, DMSO, δ (ppm)): δ 9.51 (s, H_8_), 7.46 (d, H_1_), 7.45 (d, H_5_), 3.90 (s, H_7_), 3.80 (s, H_10_).

^13^C NMR (400 MHz, DMSO, δ (ppm)): δ 166.09 (s, C_9_), 148.88 (s, C_3_), 147.47 (s, C_2_), 125.40 (s, C_5_), 124.36 (s, C_6_), 119.48 (s, C_4_), 110.92 (s, C_1_), 56.01 (s, C_7_), 51.79 (s, C_10_).

***Procedure for methylation***

26 mmol of divanillin and 15,2 g of potassium carbonate (110 mmol) were dissolved in 120 mL of DMF. 9,6 ml of iodomethane (158 mmol) were slowly added to the mixture. After 15 h at 80 °C, mixture was filtered and the resulting solution poured into cold water. The methylated compound, which precipitated was filtered off and dried under vacuum. Yield: 80%.


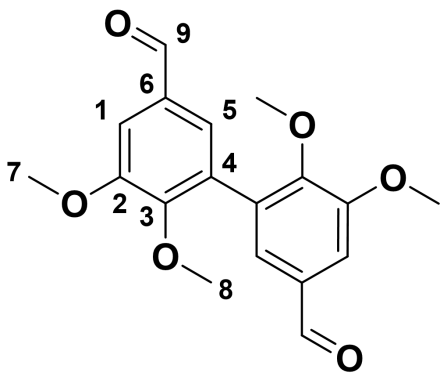


^1^H NMR (400 MHz, DMSO, δ (ppm)): δ 9.94 (d, H_9_), 7.58 (d, H_1_), 7.45 (d, H_5_), 3.95 (s, H_7_), 3.67 (s, H_8_).

^13^C NMR (400 MHz, DMSO, δ (ppm)): δ 191.76 (s, C_9_), 152.88 (s, C_2_), 151.52 (s, C_3_), 131.81 (s, C_6_), 131.56 (s, C_4_), 126.09 (s, C_5_), 111.36 (s, C_1_), 60.43 (s, C_8_), 55.99 (s, C_7_).

The same procedure was applied for methyl divanillate. Yield: 80%


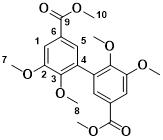


^1^H NMR (400 MHz, CDCl3, δ (ppm)): δ 7.62 (d, H_1_), 7.57 (d, H_5_), 3.95 (s, H_7_), 3.88 (s, H_10_), 3.71 (s, H_8_).

^13^C NMR (400 MHz, CDCl3, δ (ppm)): δ 166.79 (s, C_9_), 152.54 (s, C_3_), 151.06 (s, C_2_), 131.87 (s, C_6_), 125.30 (s, C_4_), 125.10 (s, C_5_), 113.09 (s, C_1_), 60.95 (s, C_8_), 56.14 (s, C_7_), 52.25 (C_10_).

***Procedure for ester hydrolysis***

10 mmol of methylated diester were dissolved in 30 mL of methanol. 3g of sodium hydroxide (75 mmol) were slowly added to the mixture and warmed to reflux during 4h. After cooling at room temperature, the solution is acidified with HCl to pH=3. The precipitate was filtered off and the product dried overnight at 80 °C under vacuum. Yield: 90%


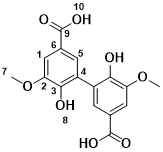


^1^H NMR (400 MHz, DMSO, δ (ppm)): δ 9.39 (s, H_8_), 7.45 (d, H_1_), 7.41 (d, H_5_), 3.89 (s, H_7_).

^13^C NMR (400 MHz, DMSO, δ (ppm)): δ 167.18 (s, C_9_), 148.36 (s, C_3_), 147.22 (s, C_2_), 125.44 (s, C_6_), 124.19 (s, C_4_), 120.44 (s, C_5_), 111.05 (s, C_1_), 55.89 (s, C_7_).

***Procedure for oximation***

1 g of hydroxylamine hydrochloride (7 mmol) and 2 g of sodium acetate (12 mmol) were solubilised in 20 mL of ethanol (+4 mL of water). 2 g of divanillin or methylated divanillin (6 mmol) were added to the mixture. After 2 h of magnetic stirring at 100 °C, the product is extracted with dichloromethane and washed with water and dried under vacuum. Yield: 85%


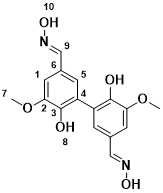


^1^H NMR (400MHz, DMSO, δ (ppm)): δ 10.83 (s, H_10_), 8.84 (s, H_8_), 8.02 (s, H_9_), 7.18 (s, H_4_), 6.94 (s, H_5_), 3.85 (s, H_7_).

^13^C NMR (400MHz, DMSO, δ (ppm)): δ 148.00 (s, C_9_), 147.62 (s, C_2_), 145.48 (s, C_3_), 125.42 (s, C_6_), 123.58 (s, C_4_), 123.02 (s, C_5_), 107.21 (s, C_1_), 56.12 (s, C_7_).


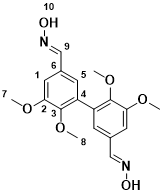


^1^H NMR (400MHz, DMSO, δ (ppm)): δ 11.58 (s, H_10_), 8.10 (s, H_9_), 7.30 (d, H_1_), 6.98 (d, H_5_), 3.87 (s, H_7_), 3.56 (s, H_8_).

^13^C NMR (400MHz, DMSO, δ (ppm)): δ 152.66 (s, C_2_), 147.81 (s, C_9_), 147.25 (s, C_3_), 131.87 (s, C_6_), 128.69 (s, C_4_), 121.69 (s, C_5_), 108.78 (s, C_1_), 59.88 (s, C_8_), 55.6 (s, C_7_).

***Procedure for reduction of oxime***

1 g of methylated divanillyloxime (2,7 mmol) and 1 mL of nickel Raney (slurry) were added in 30 mL of ethanol. The mixture was set into pressurized reactor with 10 bars of dihydrogen. After 15 h at 70 °C, the mixture was filtered and ethanol was removed under vacuum. The resulting product is extracted with dichloromethane and washed with water. Dichloromethane was removed from the organic phase under vacuum. Yield: 70%


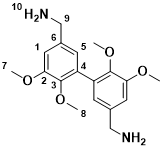


^1^H NMR (400MHz, DMSO, δ (ppm)): δ 7.04 (m, H_5_), 6.69 (m, H_1_), 3.79 (m, H_8_), 3.63 (s, H_7_), 3.48 (m, H_9_).

^13^C NMR (400MHz, DMSO, δ (ppm)): δ 151.75 (s, C_2_), 144.46 (s, C_3_), 136.04 (s, C_6_), 132.36 (s, C_4_), 121.78 (s, C_5_), 111.41 (s, C_1_), 60.05 (s, C_8_), 55.65 (s, C_7_), 51.62 (s, C_9_).

***Procedure for hydroxamic acid synthesis from ester***

1,4 g of hydroxylamine hydrochloride were solubilized in 10 mL of MeOH and 2,3g of potassium hydroxide were dissolved in 10 mL of MeOH. Both preparations were cooled down in ice, and the alkali solution was added to the hydroxylamine solution under stirring. The precipitated salts were removed by filtration and the filtrate was added to 2g of methylated diester. Additional potassium hydroxide was added to increase the pH>10 and the mixture was stirred during 12 hours at room temperature. An aqueous solution of HCl (2M) was then added to the mixture and precipitation occurred. The solid was filtered off and washed with water.

***Procedure for hydroxamic acid synthesis from oxime***

1 g of methylated divanillyloxime (3 mmol) and 2,3 g of HTIB (6 mmol) were solubilized in 2 mL of DMSO and stirred at 90°C. After 24h, 0,8 g of sodium hydroxide was added to the mixture and stirred during 96h. Finally, the reaction mixture was cooled down to room temperature and 5 mL of aqueous solution of HCl (5M) was added. Precipitation occurred and the solid was filtered off and washed with water.

***Procedure for acyl azide synthesis***

3 mmol of methylated divanillic acid were solubilized into mixture of 15mL of THF and 5 mL of water. The reaction mixture was cooled to 0 °C and 2,4 mL of triethylamine in 4 mL of THF was added drop-wise. 1,8 mL of ethylchloroformate was then added to the mixture and stirred during 2h at 0 °C. A solution of sodium azide (1,2g in 4 mL of water) was added drop-wise into the reaction mixture and stirred at 0 °C for 2 h and then at room temperature for 8 h. Cold water was added gradually to the reaction mixture to precipitate the solid. The precipitate was filtered off. The product was then dissolved in DCM, washed with water and dried over anhydrous magnesium sulfate. Dichloromethane was removed from the organic phase under vacuum. Yield: 60%


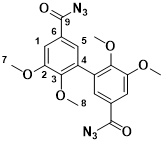


^1^H NMR (400 MHz, CDCl3, δ (ppm)): δ 7.61 (d, H_1_), 7.55 (d, H_5_), 3.96 (s, H_7_), 3.74 (s, H_8_).

^13^C NMR (400 MHz, CDCl3, δ (ppm)): δ 171.85 (s, C_9_), 152.79 (s, C_3_), 152.39 (s, C_2_), 131.69 (s, C_6_), 125.81 (s, C_4_), 125.23 (s, C_5_), 112.78 (s, C_1_), 61.10 (s, C_8_), 56.22 (s, C_7_).

***Procedure for isocyanate synthesis***

Into a Schlenk tube under nitrogen atmosphere, 0.5 mmol of diazide were solubilized in 3 mL of dry toluene and stirred. The reaction mixture was heated at 80 °C for 8 h. The toluene was removed under reduced pressure at 60 °C and white oily compound was obtained. Yield: 80%


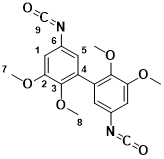


^1^H NMR (400 MHz, CDCl3, δ (ppm)): δ 6.65 (d, H_1_), 6.58 (d, H_5_), 3.88 (s, H_7_), 3.64 (s, H_8_).

^13^C NMR (400 MHz, CDCl3, δ (ppm)): δ 153.41 (s, C_3_), 144.82 (s, C_2_), 132.46 (s, C_6_), 128.72 (s, C_4_), 124.71 (s, C_9_), 118.91 (s, C_5_), 108.86 (s, C_1_), 60.99 (s, C_8_), 56.13 (s, C_7_).

***Procedure for isocyanate hydrolysis***

3 mmol of a potassium hydroxide solution were added to 0.75 mmol of di-isocyanate in solution in toluene. The mixture was stirred 12h at 80 °C. Toluene was removed under reduced pressure. The product was solubilized in ethyl acetate and washed with water. Ethyl acetate was removed from the organic phase under vacuum. Yield: <10%


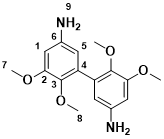


^1^H NMR (400 MHz, DMSO, δ (ppm)): δ 6.23 (d, H_1_), 5.90 (d, H_5_), 4.79 (s, H_9_), 3.72 (s, H_7_), 3.38 (s, H_8_).

^13^C NMR (400 MHz, CDCl3, δ (ppm)): δ 152.47 (s, C_3_), 144.22 (s, C_2_), 136.90 (s, C_6_), 133.35 (s, C_4_), 107.48 (s, C_5_), 98.42 (s, C_1_), 59.97 (s, C_8_), 55.18 (s, C_7_).

***Procedure for epoxy thermoset***

Epoxy monomers were mixed vigorously with stoichiometric amount (*r*=1) of IPDA, DDS, MDVA or DMAN. The mixture was then placed into an aluminum DSC pan.

**Methods**

**Nuclear Magnetic Resonance (NMR)**

All NMR experiments were performed at 298 K on a Bruker Avance 400 spectrometer operating at 400 MHz. CDCl_3_ and DMSO-d6 were used as deuterated solvent depending on the sample and specified in the legends of spectra.

**Flash chromatography**

Flash chromatography was performed on a Grace Reveleris apparatus, employing silica cartridges from Grace and a dichloromethane/methanol gradient solvent equipped with ELSD and UV detectors at 254 and 280 nm.

**Differential Scanning Calorimetry (DSC)**

Differential Scanning Calorimetry (DSC) measurements were performed on DSC Q100 (TA Instruments). The sample was heated at a rate of 10 °C.min^-1^. Consecutive cooling and second heating run were also performed at 10 °C.min^-1^. The glass transition temperatures and melting points were calculated from the second heating run.

**Dynamic Mechanical Analysis (DMA)**

Dynamic Mechanical Analysis (DMA) measurements were performed on DMA-RSA3 system from TA instruments. The three point bending sample (width = 2 mm; thickness = 2 mm and length of fixed section = 10 mm) was heated from 25 °C to 350 °C at a heating rate of 5 °C.min^- 1^. The measurements were performed in a three-point bending mode at a frequency of 1 Hz, an initial static force of 0.5 N and a strain sweep of 0.01 %.

**Thermogravimetric analyses (TGA)**

Thermogravimetric analyses (TGA) were performed on TGA-Q50 system from TA instruments at a heating rate of 10 °C.min^-1^ from room temperature to 950 °C. The analyses were investigated under air and nitrogen atmosphere with platinum pans.

**Fourier Transformed Infra-Red-Attenuated Total Reflection (FTIR-ATR)**

Infrare spectra were performed on a Bruker VERTEX 70 spectrometer, equipped with diamond crystal (GladiATR PIKE technologies) for attenuated total reflection mode. The spectra were acquired from 400 to 4000 cm^-1^ at room temperature using 32 scans at a resolution of 4 cm^-1^.


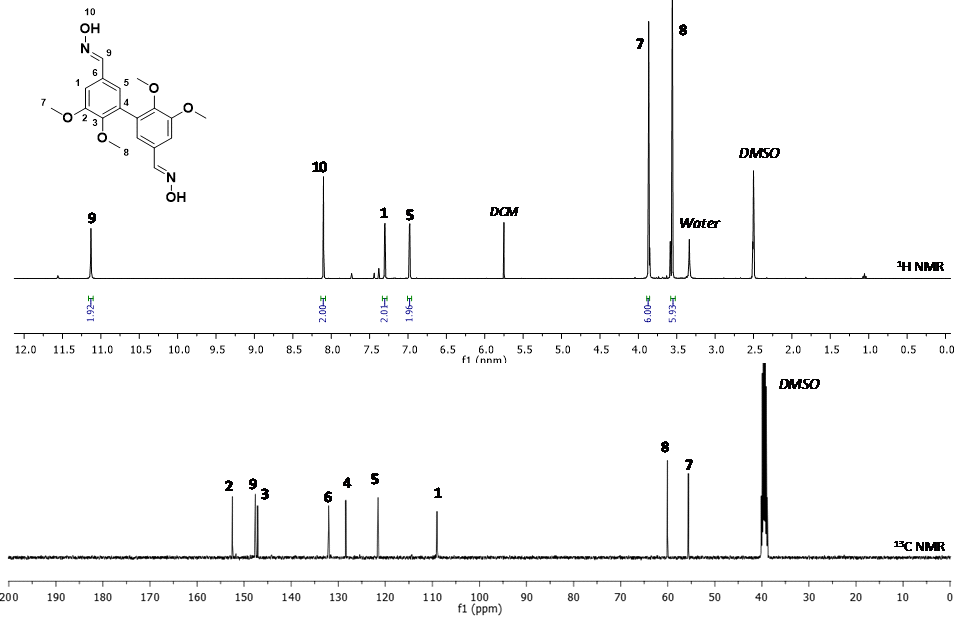


Figure S1: ^1^H and ^13^C NMR spectra of methylated divanillyloxime in DMSO-d6


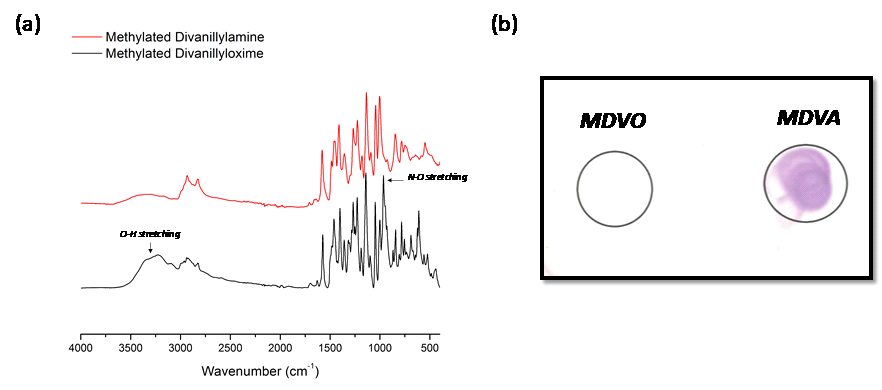


Figure S2: (a) FTIR spectra of MDVA and MDVO, (b) Ninhydrin test results of MDVO and MDVA on gel silica plate


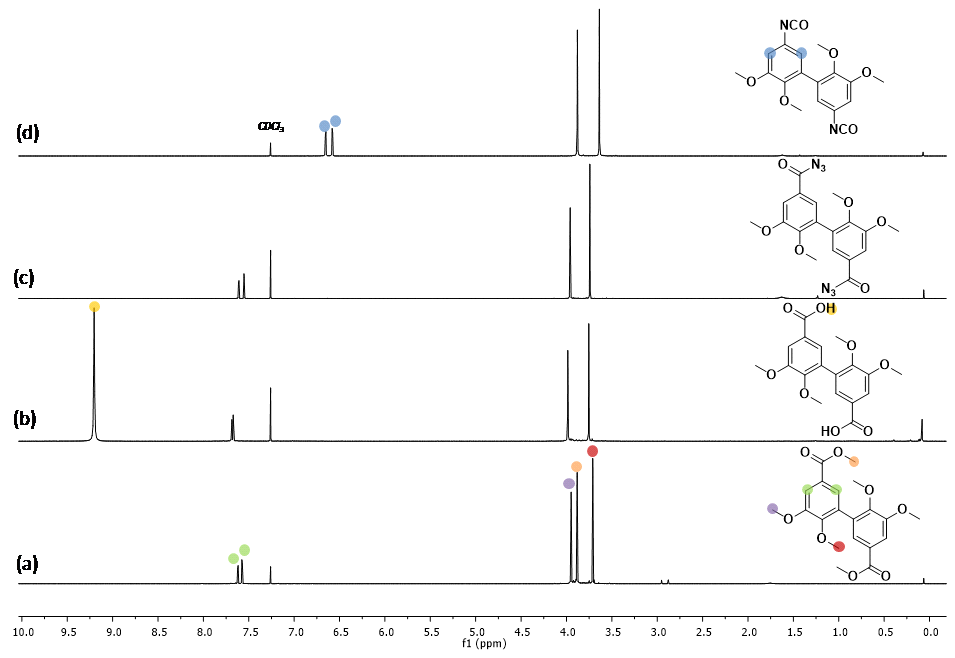


Figure S3: ^1^H NMR spectra of (a) methylated diester, (b) methylated divanillic acid, (c) methylated di-acyl azide and (d) methylated di-isocyanate of vanillin in CDCl_3_


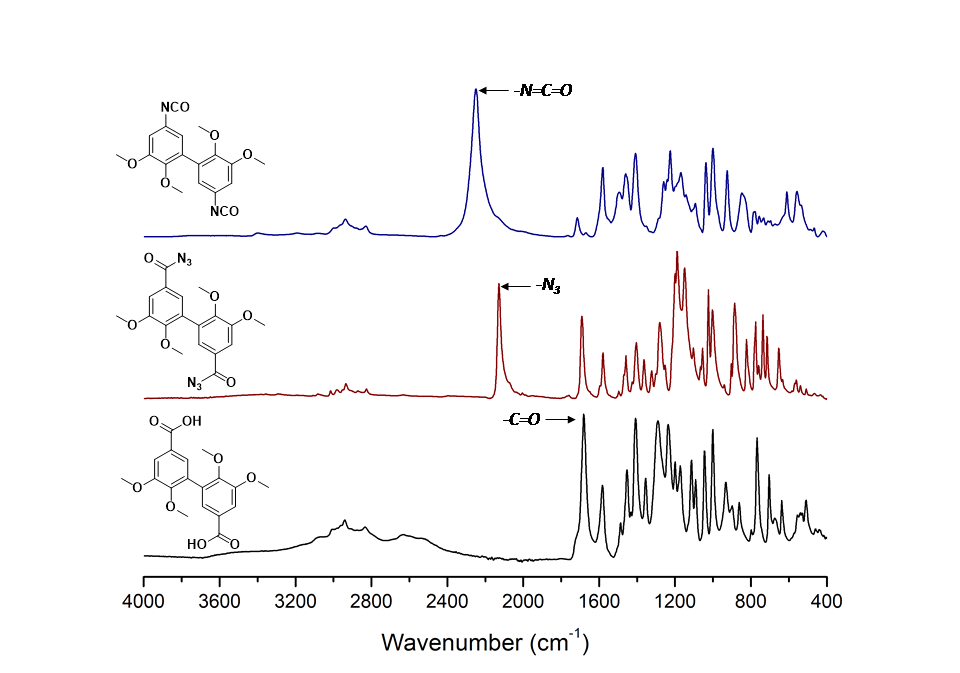


Figure S4: FTIR spectra of methylated divanillic acid, di-acyl azide and di-isocyanate


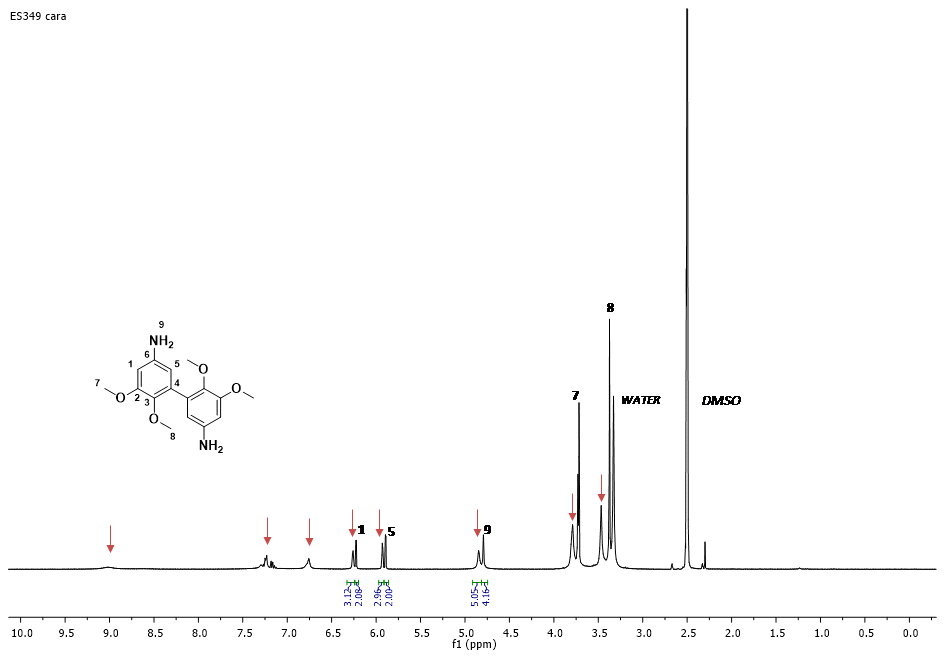


Figure S5: ^1^H spectrum of 3,4-dimethoxydianiline before purification in DMSO-d6


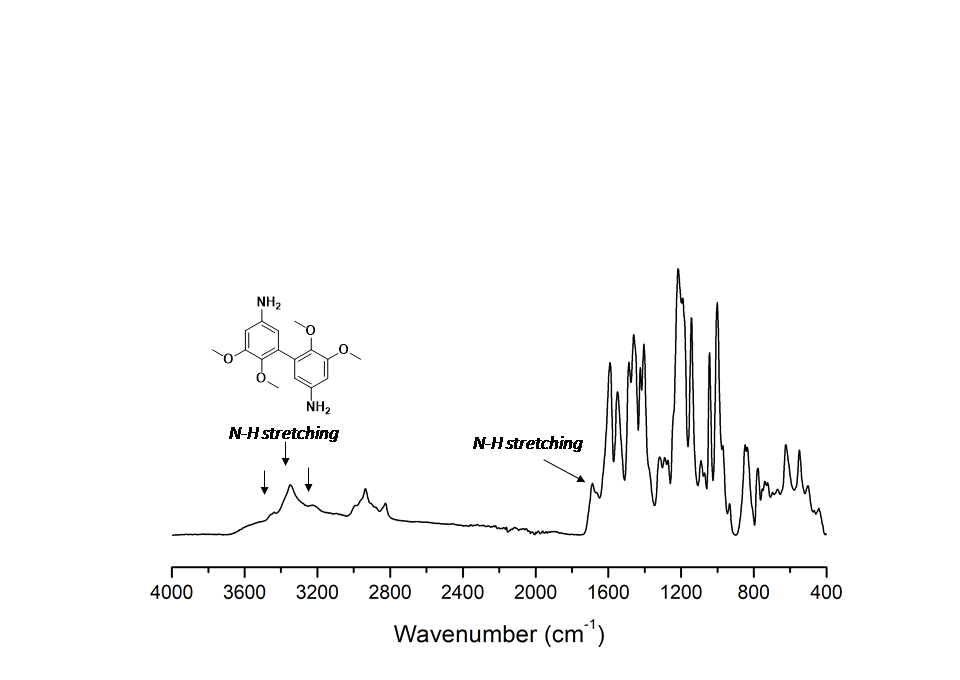


Figure S6: FTIR spectrum of 3,4-dimethoxydianiline

| Epoxy/hardener | T_Onset_  (°C) | T_Exotherm_  (°C) | ΔH  (J.g^-1^) | Tg  (°C) | Char_900_ (%) |
| --- | --- | --- | --- | --- | --- |
| DGEBA/IPDA | 73 | 111 | 430 | 152 | 8 |
| DGEBA/MDVA | 96 | 142 | 105 | * | nd |

**No Tg was clearly observed by DSC*

Table S1: Thermomechanical properties of DGEBA cured with IPDA and MDVA


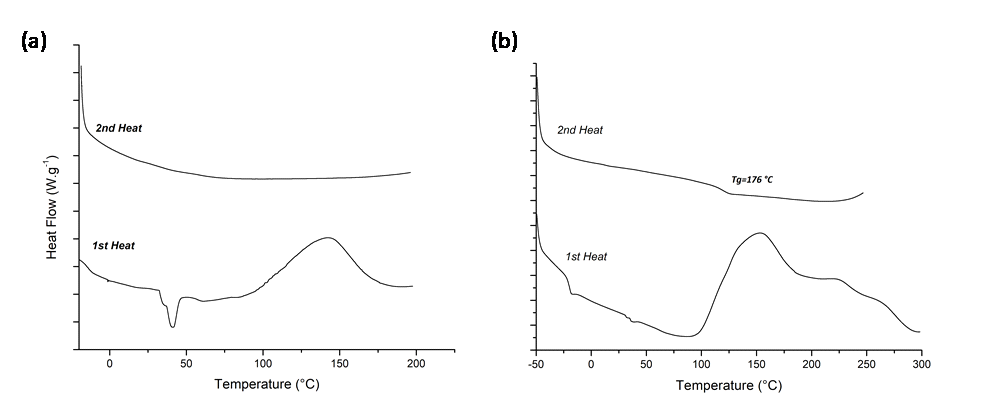


Figure S7 : DSC thermograms of (a) DGEBA/MDVA and (b) DGEBA/DMAN


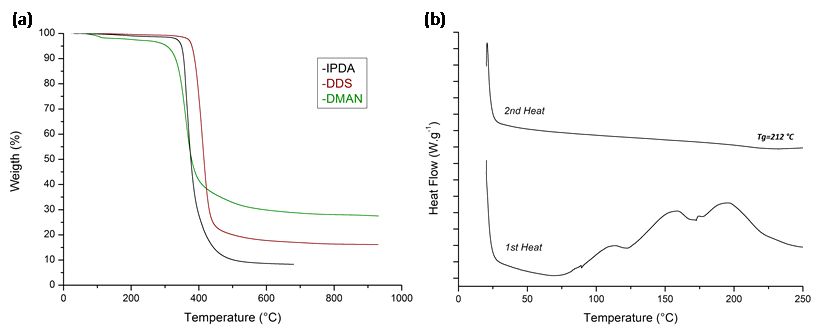


Figure S8: (a) TGA thermograms of DGEBA cured with IPDA, DDS and DMAN, (b) DSC thermograms of TetraGEDVA cured with DMAN

1. E. Savonnet, E. Grau, S. Grelier, B. Defoort and H. Cramail, *ACS Sustain. Chem. Eng.*, 2018, **6**, 11008–11017. [↑](#footnote-ref-1)
